# A Self-Adaptive Parameter Optimization Algorithm in a Real-Time Parallel Image Processing System

**DOI:** 10.1155/2013/978548

**Published:** 2013-09-23

**Authors:** Ge Li, Xuehe Zhang, Jie Zhao, Hongli Zhang, Jianwei Ye, Weizhe Zhang

**Affiliations:** ^1^State Key Laboratory of Robotics and System, Harbin Institute of Technology, No. 2, Yikuang Street, Harbin 150001, China; ^2^School of Computer Science and Technology, Harbin Institute of Technology, No. 92, West DA-Zhi Street, Harbin 150001, China

## Abstract

Aiming at the stalemate that precision, speed, robustness, and other parameters constrain each other in the parallel processed vision servo system, this paper proposed an adaptive load capacity balance strategy on the servo parameters optimization algorithm (ALBPO) to improve the computing precision and to achieve high detection ratio while not reducing the servo circle. We use load capacity functions (*LC*) to estimate the load for each processor and then make continuous self-adaptation towards a balanced status based on the fluctuated *LC* results; meanwhile, we pick up a proper set of target detection and location parameters according to the results of *LC*. Compared with current load balance algorithm, the algorithm proposed in this paper is proceeded under an unknown informed status about the maximum load and the current load of the processors, which means it has great extensibility. Simulation results showed that the ALBPO algorithm has great merits on load balance performance, realizing the optimization of QoS for each processor, fulfilling the balance requirements of servo circle, precision, and robustness of the parallel processed vision servo system.

## 1. Introduction

Image processing often requires large-scale data, and many application fields require real-time performance. Thus, image parallel processing systems have been widely applied in the field of image processing to meet the needs of practical applications as discussed by Plaza et al. [1]. However, the actual performance of image processing is often lower than the peak performance provided by this system, implying that the design of the parallel algorithm is essential for improving system performance as discussed by Krefting et al. [2]. An efficient parallel algorithm should enable the load to achieve balance, evenly distribute tasks, and minimize the amount of communication between nodes and thus improving the performance of the parallel treatment system as discussed elsewhere [3, 4]. Therefore, the load balancing scheduling strategy is a difficult and enticing area of research in parallel processing.

In general, the load balancing scheduling strategy is divided into static and dynamic strategies. The static information of the system is used in the load balancing strategy when the load is distributed on each node, making it easy to be analyzed and implemented. However, such a load balancing strategy results in low utilization factor and performance degradation for it neglects the variations in the node load as discussed by Tantawi and Towsley [5]. On the other hand, the dynamic load balancing strategy can dynamically determine load migration according to the system load at each moment, improving system performance significantly as discussed by Ren et al. [6]. Current studies on the scheduling algorithm have been generally committed to seeking a better suboptimal scheduling algorithm. In particular, the self-adaptive load balancing scheduling strategy has been widely considered among professional researchers due to its ability for automatic adjustment in load balancing based on the information state of the system as discussed by Porter and Katz [7]. In addition, the self-adaptive load balancing scheduling strategy improves the load balancing effect through the perception of load variation. The load balancer collects load information on each node periodically and then utilizes the load information to determine the available node that can directly service the customer's request. The most common self-adaptive load balancing scheduling strategy includes strategies based on the minimum as discussed by Baumgartner et al. [8] and average loads as discussed by Bhatelé et al. [9]. 

This study focuses on the parallel processing of a robotic stereoscopic vision servo system. According to the scheme of the parallel partition of system and the methodology of image processing, a self-adaptive load balancing scheduling strategy based on the load capacity (*LC*) measure is presented to achieve the optimization of the system among the servo cycle, servo accuracy, and system robustness.

## 2. Adaptive Load Balancing Algorithm Based on Load Capacity Measure

The *LC* measure is the estimate of the processor load, and it can maintain self-adaptive adjustment according to the information given by processor load fluctuation to achieve the optimal quality of service (QoS) of each processor. The basic principle of a self-adaptive load balancing algorithm based on the *LC* measure is that each processing node utilizes an *LC* measure to represent the current load status of the nodes. Moreover, the value of *LC* measure can undergo continuous self-adaptive adjustment according to the state of processor load fluctuation. Consequently, the system can achieve load balance depending on the suitable combination of parameters selected through the value of *LC*.

### 2.1. Problem Description

The parameters of target detection and location algorithm for the control should be adjusted in the process of program run to improve the performance of a visual servo system and achieve balance among the servo cycle, servo accuracy, and system robustness.

In the adjustable target detection and location algorithm, a number of parameters can be set for the control of the performance of image processing. For example, *λ*_*c*_ represents the interval length of the color image segmentation threshold, *λ*_*g*_ represents the searching threshold length of the template matching projection, and *λ*_*s*_ represents the interval length that needs to be matched in the stereo matching algorithm. The control parameters of image processing are normalized as follows to uniformly evaluate the adjustment parameters of parallel processing, as in

(1) $$\begin{array}{c} \bar{\lambda_{c}}=\frac{\left(\lambda_{c}-\lambda_{\min}^{c}\right)}{\left(\lambda_{\max}^{c}-\lambda_{\min}^{c}\right)},\\ \bar{\lambda_{g}}=\frac{\left(\lambda_{g}-\lambda_{\min}^{g}\right)}{\left(\lambda_{\max}^{g}-\lambda_{\min}^{g}\right)},\\ \bar{\lambda_{s}}=\frac{\lambda_{s}-\lambda_{\min}^{s}}{\lambda_{\max}^{s}-\lambda_{\min}^{s}}, \end{array}$$

where *λ*_max⁡_^*i*^ is the maximum value of *λ*_*i*_,  *λ*_min⁡_^*i*^ is the minimum value of *λ*_*i*_, and *i* ∈ {*c*, *g*, *s*}.

*M*_*c*_ was assumed to be the number of color image processing results. *M*_*g*_ is the number of template matching processing results. *M*_*s*_ is the number of first step results in stereo matching processing. The load fluctuation rate Δ_*j*_ of processor *j* is defined as the degree of change in the load state parameters of system output in cases where devices do not work and the control parameters in charge of load balance are invariant. In the current study, the system load fluctuation rate Δ_*j*_ includes the fluctuation rate of color image processing Δ_*c*_^*j*^, the template matching processing Δ_*g*_^*j*^, and the stereo matching processing Δ_*s*_^*j*^. The threshold processing of the image processing algorithm used in this system satisfies the normal distribution. Therefore, *λ*_*s*_^*j*^ has an exponential relationship to *M*_*i*_^*j*^, and the calculating formula of the fluctuation rate can be defined as follows:

(2) $$\begin{array}{c} \Delta_{i}^{j}=K_{i}^{j}\left(k\right)\frac{M_{i}^{j}\left(k\right)-M_{i}^{j}\left(k-1\right)}{M_{i}^{j}\left(k\right)+M_{i}^{j}\left(k-1\right)}, \end{array}$$

where *K*_*i*_^*j*^(*k*) is a scale coefficient that relates to *M*_*i*_^*j*^(*k*) and the data size of processing algorithm *i* and meets the condition of *K*_*i*_^*j*^(*k*) = 1/ln⁡(*M*_*i*_^*j*^(*k*)).

In terms of the calculating formula of the fluctuation rate, Δ_*j*_^*i*^ is deemed equal to a number from −1 to 1 and only needs to be calculated by counting the number of computed results of the image processing algorithm when the processor is running in unit cycle, which is a very simple statistical process.

### 2.2. Load Capacity

*LC* is a measure of the calculated amount of the current processor that can be accommodated, and it can be used to estimate the relative load status of each processing node. A smaller *LC*_*j*_(*k*) indicates the smaller capacity of the processor; that is, the current processor has a smaller calculating load and can contain more computations. The admission processing should be decreased when *LC*_*j*_(*k*) is greater than 1 because of the heavy processor load. The values of *LC*_*j*_(*k*) can continuously execute self-adaptive adjustment according to the situation of load fluctuation, adjustment parameters, and the status of image acquisition:

(3) LCj(k)=(1+fj(statej−6,…,statej)) ×∑i∈Wjβij(k)LCij(k),

(4) $$\begin{array}{c} \sum_{i\in W_{j}}\beta_{i}^{j}\left(k\right)=1\;\beta_{i}^{j}\left(k\right)⩾0, \end{array}$$

where *W*_*j*_ is a collection of all adjustable algorithms of image processing that can be included by processor *j*.

In (3), *f*_*i*_(state_*j*−6_,…, state_*j*_) represents a capacity evaluation function; that is, a conditions function. *f*_*i*_(state_*j*−6_,…, state_*j*_) can be expressed as follows:

(5) $$\begin{array}{c} f_{j}\left(\text{stat}{\text{e}}_{j-5},\ldots ,\text{stat}{\text{e}}_{j}\right)=\frac{\left(\gamma_{f}\cdot \sum_{i=j-5}^{j}\text{stat}{\text{e}}_{i}\cdot \left|\sum_{i=j-5}^{j}\text{stat}{\text{e}}_{i}\right|\right)}{36}, \end{array}$$

where *γ*_*f*_ is the adjustment range factor.

The capacity rate evaluation function is used for judging the capacity size of processor settings. The capacity rate evaluation function uses the sources of processor data to determine the overall increase or decrease in the load capacity rate. In other words, the processing speed is considered too slow when the processor has completed the assignments in a servo cycle and the original data of next servo cycle has been in the accepted state (state_*j*_ = 1), causing the servo cycle of the processor to be longer than the desired system cycle (40 ms). Therefore, the oversize setting of *LC* should be reduced. On the other hand, the processing speed is considered too fast and the servo cycle of processor is shorter than the desired system cycle (40 ms) when the data has not been received (represented by zero). In this situation, the settings of the total *LC* rate are too small and should be increased.

In (3), the initial value of algorithm *i* in the specific weight *β*_*i*_^*j*^(*k*) is set as *β*_*i*_^*j*^(0) = 1/*m*_*b*_^*j*^, where *m*_*b*_^*j*^ represents the number of adjustable image processing parameters that move under processor *j*. In other words, all reliabilities of the adjustable processing algorithms and weights in the process are deemed equal during initialization. However, the reliabilities of the adjustable algorithms are different in practical applications because the adjustment of weight parameters in each algorithm must be calculated using the other parameters following different parallel processing architectures. In this study, the adjustment of weight in a visual servo parallel processing system is executed between the color image processing and the template matching, which is primarily affected by the target's changes in shape. As can be seen in (6), the change in rate of the target's shape *θ*_*p*_(*k*) is defined as the proportion of the trace tr⁡(*R*(*k*)) of rotation transformation between the current target and the template when the degrees of change in the shape of the objective are evaluated. Furthermore, the threshold value tr⁡(*R*_*m*_) of the trace of rotation transformation is executed when the template must be updated as follows:

(6) $$\begin{array}{c} \theta_{p}\left(k\right)=\frac{3-\mathrm{tr}\left(R\left(k\right)\right)}{3-\mathrm{tr}\left(R_{m}\right)}. \end{array}$$

Then, the specific weight *β*_*g*_^*j*^(*k*) and *β*_*c*_^*j*^(*k*) can be expressed as follows:

(7) $$\begin{array}{c} \beta_{g}^{j}\left(k\right)=\gamma_{p}\theta_{p}\left(k\right),\\ \beta_{c}^{j}\left(k\right)=1-\beta_{g}^{j}\left(k\right), \end{array}$$

where *γ*_*p*_ is the rotation adjustment factor and is a constant between 0 and 0.5.

### 2.3. Adjustment and Update Strategy

The adjustment capacity of the current capacity *LC_*i*_^*j*^(*k*) is based on the evaluation function of capacity, the weight of the algorithm, and the computing formulae of *LC* as follows:

(8) LCij∗(k)=LCij(k)βij(k)LCj(k)βij(k−1)LCj(k−1).

The new *LC* can be calculated via the fluctuation ratio Δ_*i*_^*j*^ and adjustment parameters after the new state parameters are obtained. The new *LC* can be expressed as follows:

(9) LCij(k)=(1+αΔij)(1+λ¯ij(k)−λ¯ij(k−1))LCij∗(k−1),

where *α* is the fluctuation factor, that is, constant between 0 and 1, reflects the magnitude of acutely adjusting *LC*_*j*_(*k*) along with load fluctuation.

The first and middle multiplicative factors on the right-hand side of (9) represent the effect of the fluctuation ratio and the effect of the variable adjustment parameter on *LC*, respectively. The fluctuation factor *α* weakens the effect of load fluctuation ratio on *LC* when the load of the processor is progressively varied with time.

Based on the previous theory, a function for updating control parameters can be established as follows:

(10) $$\begin{array}{c} {\bar{\lambda }}_{i}^{j}\left(k+1\right)=1-LC\left(k\right)+{\bar{\lambda }}_{i}^{j}\left(k\right). \end{array}$$

Equation (10) reveals that the scale of the control parameters slowly decreases when the load capacity *LC*_*i*_^*j*^(*k*) approaches unity, avoiding the occurrence of the load thrashing phenomenon. The processor rapidly achieves a balance among the servo cycle, the servo precision, and system robustness because the control parameters have become capable of rapid increases due to the minimal load, thereby achieved the optimization of each processing QoS.

## 3. Application Characteristic of ALBPO Algorithm 

### 3.1. Properties of ALBPO Algorithm

The entire system was assumed to contain *n* parallel image processing subsystems. Without loss of generality, the single system was assumed to have *m*_*i*_ performance-adjustable parameters, and the performance-adjustable parameters *λ*_*i*_^*j*^ in each algorithm *F*_*i*_^*j*^ should exhibit the following properties.

1. For the same image, the computational complexity of the algorithm *F*_*i*_^*j*^ increases when the parameters *λ*_*i*_^*j*^ increase, improving the calculation accuracy and robustness of image processing. On the other hand, the computational complexity of algorithm *F*_*i*_^*j*^ decreases when the parameters *λ*_*i*_^*j*^ decrease, reducing the calculation accuracy and robustness of image processing.
2. For the same image, portions of the computation randomly fluctuate in certain areas when the parameters *λ*_*i*_^*j*^ are constant due to the images are corrupted with noise.
3. The parameters *λ*_*i*_^*j*^ occur via the normalization process, and thus their values are {0, 1}.

Moreover, the image parallel processing system has difficulty in accurately evaluating the processor's maximum computation during the image parallel process.

### 3.2. Initial Settings of ALBPO Algorithms

The following initial settings were established according to the properties of the ALBPO algorithm.

1. The initial values of the performance-adjustable parameters *λ*_*i*_^*j*^ in the *F*_*i*_^*j*^ algorithm were set such that they allow large deviations.
2. At the initial time and all prior times, *LC*_*j*_ = 1, the load capacities *LC*_*i*_^*j*^ = 1, and the product of adjustable capacity rate *LC_*i*_^*j*^ = 1 in each algorithm *F*_*i*_^*j*^.
3. At the initial time and all prior times, the receiving status of each processor State_*k*_ = 0, and *k* ∈ {−5, −4, −3, −2, −1,0}.

## 4. Experimental Studies

### 4.1. Experimental System

This study conducted simulation experiments to verify the optimized performance of the ALBPO algorithm for visual servo adjustment parameters and to achieve balance among the servo cycle, servo accuracy, and system robustness in the parallel processing visual servo system. This problem can be attributed to the search for a set of maximum *λ*_*i*_^*j*^, making the receiving status values of each processor frequently switch between −1 and 1. The simulation on a single processor can be verified in the property of the ALBPO algorithm that is independent in each processor. The *F*_*i*_^*j*^ algorithms, which run on each processor, exhibit identical properties, and thus the corresponding situation can be used to verify which processor has two performance-adjustable parameters.

The total computational amount *Ω*_max⁡_ of the system was assumed to be known and constant to verify the performance of the ALBPO algorithm. The performance-adjustable parameter *λ*_1_ and the computational amount *Ω*_1_ were assumed to exhibit a linear relationship, *Ω*_1_ = *k*_1_ × *λ*_1_. The performance-adjustable parameter *λ*_2_ and the computational amount *Ω*_2_ were assumed to exhibit a nonlinear relationship, *Ω*_2_ = (*k*_2_ + *k*_2_ × *λ*_2_) × *λ*_2_. Both algorithms exhibit the random disturbance quantity.

### 4.2. Parameter Optimization Results of ALBPO Algorithm

**Figure 1:**
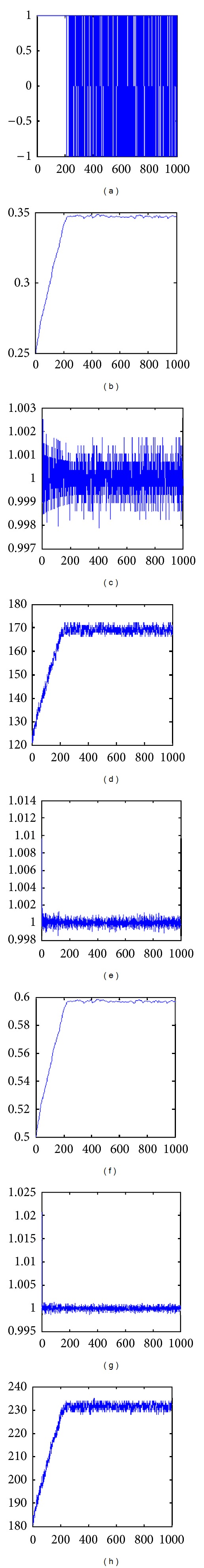
The monitoring data of the optimization results for the ALBPO algorithm.

**Figure 2:**
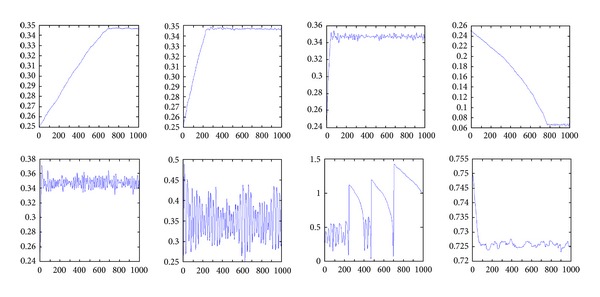
The optimization results for different *γ*_*f*_ and *α*.

Assume that *Ω*_max⁡⁡_ = 400, *λ*_1_ = 0.25, *λ*_2_ = 0.5, Λ = 5, *k*_T1_ = 480, *k*_T2_ = 240, *γ*_*f*_ = 1, *α* = 0.6, and servo cycle *T* = 1000.  Figure 1 shows the effects of the parameter optimization results of the SALB algorithm. The horizontal coordinates represent the sampling number. The vertical coordinates represent different values in the following different figures.  Figure 1(a) represents the value of the state in the capacity rate evaluation function. Figures 1(b), 1(c), and 1(d) represent the number of processing results, capacity rate, and values of adjustable parameters, respectively.  Figure 1(e) shows the overall capacity rate of the processor. Figures 1(h), 1(g), and 1(f) represent the number of processing results, capacity rate, and values of adjustable parameters, respectively. The first flip in state (state = −1) when the sampling size was 214 indicates that the initial data of the next servo cycle has changed and is awaiting the receiving state in the already received state, as shown in Figure 2. The subsequent state-repeated shocks indicate that the values of adjustable parameters simply satisfy the values of the maximum computational amount achieved by the processor. Moreover, Figures 1(b) and 1(f) indicate that the adjustable parameters of the ALBPO algorithms monotonically increase first and then remain unchanged, and thus, the adjustable parameters can be adjusted using the ALBPO algorithm to achieve the optimal control point. Changes in the number of the processing results also confirm the adjustment effect of the ALBPO algorithm. In addition, as can be seen in Figures 1(e), 1(c), and 1(g), the overall capacity rate of the two algorithms both changed at a very low rate during processing, denoting that the system can still be regulated when the system is initializing, even if the parameters of the artificial settings present deviations.

### 4.3. Effects of Adjusting Amplitude Factor *γ*_*f*_ and Volatility Factor *α* on Optimization Results

Figure 1 shows the servo parameters optimization results of the ALBPO algorithm when *γ*_*f*_ = 1 and *α* = 0.6. The values of *γ*_*f*_ and *α* influence the optimization of the servo parameters significantly, thus properly selecting the values of *γ*_*f*_ and *α* can generate better optimization results. Therefore, investigating the connection between the servo parameters optimization results and adjusting the variations in the amplitude factor *γ*_*f*_ and volatility factor *α* are necessary. The maximum and minimum fluctuation values of the stable period of the system and the adjustment parameters in the stable state when *γ*_*f*_ and *α* are variable are shown in Table 1. Figure 2 shows the optimization results for the different values of *γ*_*f*_ and *α*.

Figure 2 also shows the optimization results for different values of *γ*_*f*_ and *α*. By analyzing the tabular data, the product size of *γ*_*f*_ and *α* was found to affect the stable period of the system, in addition to the fluctuation range of adjustment parameters in the steady state. The fluctuation range increased and the stable period decreased when the product of *γ*_*f*_ and *α* increased. Moreover, the fluctuation range decreased and the stable period increased when the product of *γ*_*f*_ and *α* decreased. Finally, the fluctuation range and the stable period exhibited minimal changes when the product of *γ*_*f*_ and *α* was constant.

## 5. Conclusions

The current study proposed a multiprocessor parallel scheduling algorithm to adjust the performance parameters of the image processing of the visual servo system. The proposed ALBPO algorithm was shown to be efficient in improving the computational accuracy and recognition rate under the condition that the visual servo system did not reduce the servo cycle. In the ALBPO algorithm, the unknown and time-varying maximum amounts of calculation of a processor were exchanged via *LC*, and the *LC* measure and parameters were adjusted by adopting the fluctuation rate of load and the evaluation function of the state. Therefore, the optimization of the QoS of each processor and the coordination among the servo accuracy, servo speed, and system robustness were easily realized. The feasibility and efficiency of the ALBPO algorithm were demonstrated through a simulation study on the parallel processing scheduling algorithm.

## Figures and Tables

**Table 1:** The effects of amplitude factor and volatility factor on system performance.

*α*	*γ*_*f*_	*T*_first_	*λ*_max⁡_^1^	*λ*_min⁡_^1^	*λ*_max⁡_^2^	*λ*_min⁡_^2^
0.6	1	231	0.3487	0.3449	0.5987	0.5949
0.3	1	443	0.3475	0.3452	0.5975	0.5952
0.2	1	650	0.3472	0.343	0.5972	0.593
0.1	1	1	0.3475	0.3452	0.5975	0.5952
0.15	1	878	0.3467	0.3441	0.5967	0.5941
1	1	133	0.3492	0.345	0.5992	0.595
2	1	49	0.3537	0.3398	0.6037	0.5898
4	1	15	0.3712	0.3311	0.6212	0.5811
7	1	12	0.4872	0.2508	0.7372	0.5008
8.4	1	10	1.4228	0.036	1.6728	0
0.6	2	92	0.3504	0.3431	0.6004	0.5931
0.6	4	43	0.3551	0.3402	0.6051	0.5902
0.6	10	12	0.3881	0.3047	0.6381	0.5547
0.6	11.5	15	1.3346	0.0976	1.5846	0.3476
0.6	0.5	439	0.3485	0.3446	0.5985	0.5946
0.6	0.25	856	0.3472	0.3442	0.5972	0.5942
1.2	0.5	214	0.3495	0.3459	0.5995	0.5959
2.4	0.25	229	0.3485	0.3457	0.5985	0.5957
0.1	6	237	0.3487	0.3453	0.5987	0.5953
0.3	0.5	850	0.3469	0.343	0.5969	0.593
1.2	2	44	0.3532	0.3385	0.6032	0.5885
1.8	3	11	0.4026	0.3143	0.6526	0.5643
